# Circulating Interleukin-11 Across Allergic and Non-Allergic Airway Disease Phenotypes: A Single-Center Exploratory Study

**DOI:** 10.3390/life16091497

**Published:** 2026-09-07

**Authors:** Corina Porr, Anca Vidrighin, Emi Marinela Preda, Gabriela Mariana Iancu, Dana M. Harris, Valentin-Cristian Iovin, Alina Camelia Catana, Cosmina Diaconu

**Affiliations:** 1 Allergology Department, Faculty of Medicine, Lucian Blaga University of Sibiu, 550169 Sibiu, Romania; corina.porr@ulbsibiu.ro; 2 Allergology Department, County Clinical Emergency Hospital Sibiu, Bulevardul Corneliu Coposu 2–4, 550245 Sibiu, Romania; 3 Department of Paediatrics, Faculty of Medicine, Lucian Blaga University of Sibiu, 550169 Sibiu, Romania; anca.vidrighin@ulbsibiu.ro; 4 Department of Radiology, Carol Davila University of Medicine and Pharmacy, 050474 Bucharest, Romania; 5 Department of Radiology and Medical Imaging, Foișor Clinical Hospital of Orthopaedics, Traumatology and Osteoarticular Tuberculosis, 021382 Bucharest, Romania; 6 Department of Dermatology, Faculty of Medicine, Lucian Blaga University of Sibiu, 550169 Sibiu, Romania; 7 Clinic of Dermatology, County Clinical Emergency Hospital Sibiu, Bulevardul Corneliu Coposu 2–4, 550245 Sibiu, Romania; 8 Department of Internal Medicine, Mayo Clinic, Jacksonville, FL 32224, USA; harris.dana@mayo.edu; 9 Doctoral School Department, Victor Babeș University of Medicine and Pharmacy of Timișoara, 300041 Timișoara, Romania; valentin.iovin@umft.ro; 10 Department III Functional Sciences, Physiology Discipline, Victor Babeș University of Medicine and Pharmacy of Timișoara, 300041 Timișoara, Romania; 11 Centre of Immuno-Physiology and Biotechnologies (CIFBIOTEH), Department of Functional Sciences, Physiology, Victor Babeș University of Medicine and Pharmacy of Timișoara, 300041 Timișoara, Romania; 12 Hematology Department, Faculty of Medicine, Lucian Blaga University of Sibiu, 550169 Sibiu, Romania; alinacamelia.catana@ulbsibiu.ro; 13 Department of Nursing and Dental Medicine, Faculty of Medicine, Lucian Blaga University of Sibiu, 550024 Sibiu, Romania; cosmina.diaconu@ulbsibiu.ro; 14 Clinic of Physical Medicine and Rehabilitation, County Clinical Emergency Hospital Sibiu, Bulevardul Corneliu Coposu 2–4, 550245 Sibiu, Romania

**Keywords:** allergic rhinitis, asthma, biomarker, interleukin-11, serum

## Abstract

Interleukin-11 (IL-11) is implicated in epithelial dysfunction, fibroblast activation, tissue remodeling, and chronic inflammatory responses; however, the clinical value of circulating IL-11 in airway disease remains uncertain. This single-center retrospective exploratory observational study included 88 adults: 31 with allergic rhinitis, 15 with non-allergic asthma, 22 with allergic asthma associated with allergic rhinitis, and 20 healthy controls. Serum IL-11 concentrations were measured using a quantitative sandwich enzyme-linked immunosorbent assay. Between-group differences were assessed using the Kruskal–Wallis test followed by Holm-adjusted pairwise Mann–Whitney U tests. Associations with disease-specific ordinal clinical categories were evaluated using Spearman rank correlation, with GINA treatment Steps used for asthma phenotypes and ARIA severity categories for allergic rhinitis. Median IL-11 concentrations were 62.01 pg/mL in non-allergic asthma, 78.87 pg/mL in allergic rhinitis, 61.92 pg/mL in allergic asthma associated with allergic rhinitis, and 95.46 pg/mL in healthy controls. The primary global comparison was not statistically significant (H = 5.667, *p* = 0.129), and no pairwise comparison remained significant after Holm correction. Six measurements (6.8%) were below the assay detection limit; replacing these values with 4.0 pg/mL in a sensitivity analysis did not materially alter the global result (H = 5.671, *p* = 0.129). Serum IL-11 was not significantly associated with GINA treatment step in non-allergic asthma (rho = −0.170, *p* = 0.544) or allergic asthma associated with allergic rhinitis (rho = 0.052, *p* = 0.818), nor with ARIA severity category in allergic rhinitis (rho = 0.046, *p* = 0.805). In an exploratory analysis adjusted for age, sex, body-mass category, and current smoking status, IL-11 concentrations were lower in each clinical phenotype relative to healthy controls; given the modest group sizes and baseline imbalance, this finding was considered hypothesis-generating. Overall, the primary analysis did not demonstrate significant differences in circulating IL-11 across study groups, and serum IL-11 was not associated with disease-specific ordinal clinical categories. These findings do not support the use of a single serum IL-11 measurement as a stand-alone biomarker of airway disease phenotype or clinical category in this cohort and warrant confirmation in larger, prospectively characterized cohorts.

## 1. Introduction

Asthma and allergic rhinitis are heterogeneous inflammatory disorders that frequently coexist and share anatomical, immunological, and functional links within the united-airways framework [1,2,3,4,5]. Despite this overlap, individual patients differ substantially in inflammatory pathways, clinical severity, structural airway changes, and treatment response [6,7,8,9,10,11]. This heterogeneity has encouraged the investigation of circulating biomarkers that might complement conventional clinical assessment and established type 2 inflammatory markers [8,12,13,14,15].

Interleukin-11 (IL-11), a member of the IL-6 cytokine family, has emerged as a mediator at the interface between epithelial injury, stromal activation, inflammation, fibrosis, and impaired tissue regeneration [16,17,18,19,20,21,22,23,24,25,26,27,28,29,30,31]. Airway epithelial cells, fibroblasts, airway smooth-muscle cells, and eosinophils can produce IL-11 after inflammatory or tissue-damaging stimuli [16,17,20,23]. Increased IL-11 expression has been demonstrated in bronchial tissue from patients with moderate-to-severe asthma, while experimental airway overexpression has been associated with lymphocytic inflammation, extracellular-matrix accumulation, subepithelial fibrosis, airway smooth-muscle alterations, and airflow obstruction [16,17].

However, biological activity within airway tissue does not necessarily imply that circulating IL-11 is a clinically informative biomarker. Serum concentrations may be influenced by systemic production, clearance, assay characteristics, comorbid conditions, and the limited transfer of locally produced cytokines into the circulation. Circulating IL-11 has been insufficiently characterized across allergic rhinitis, non-allergic asthma, combined allergic airway disease, and healthy individuals within the same clinical cohort.

The primary objective of this exploratory study was to compare serum IL-11 concentrations across three airway disease phenotypes and healthy controls. A secondary objective was to examine whether circulating IL-11 was associated with disease-specific ordinal clinical categories within each phenotype. Given the established tissue-level involvement of IL-11 in airway inflammation and remodeling, we sought to determine whether these biological effects were reflected by measurable differences in circulating IL-11 concentrations.

## 2. Materials and Methods

### 2.1. Study Design and Participants

This single-center retrospective observational comparative study evaluated 88 adults assessed between January 2025 and December 2025 at the Allergy Department of the County Clinical Emergency Hospital Sibiu, Romania. The retrospective design refers to the analysis of archived clinical data and previously collected serum samples. Participants were identified among hospitalized patients and individuals evaluated in the specialized outpatient clinic.

Participants were classified into four groups: allergic rhinitis (*n* = 31), non-allergic asthma (*n* = 15), allergic asthma associated with allergic rhinitis (*n* = 22), and healthy controls (*n* = 20). All participants were adults, and written informed consent had been obtained before inclusion in the study.

### 2.2. Eligibility and Clinical Phenotyping

Adults were eligible when the available records documented a confirmed diagnosis of allergic rhinitis or asthma, disease duration of at least 2 years, and fulfillment of the disease-specific diagnostic criteria applicable during the study period. Pregnant women, patients with severe chronic systemic diseases, and individuals with alternative causes of rhinitis or asthma were excluded. Healthy controls were recruited from individuals evaluated within the same institutional setting during the study period who were clinically healthy at inclusion and had no known history of allergic airway disease, asthma, autoimmune disease, or chronic inflammatory disease. Because the control group was retrospectively identified, a uniform prospective screening protocol including standardized respiratory symptom assessment, spirometry, FeNO, or systematic allergen sensitization testing was not available for all controls. Therefore, subclinical or undocumented airway disease or atopy cannot be completely excluded.

Allergic airway disease was defined by a compatible clinical diagnosis together with documented IgE-mediated sensitization demonstrated by positive skin prick testing to clinically relevant aeroallergens. Allergic asthma associated with allergic rhinitis was defined by the coexistence of asthma and allergic rhinitis with demonstrable aeroallergen sensitization. Non-allergic asthma was defined as physician-diagnosed asthma in the absence of sensitization to the common aeroallergens included in the skin prick test panel. The non-allergic asthma group was not further classified according to eosinophilic or neutrophilic inflammatory endotype because complete peripheral blood eosinophil counts and airway inflammatory cell profiles were not consistently available in the archived dataset. Total IgE and peripheral blood eosinophil counts were not used as mandatory criteria for group allocation.

Asthma diagnosis and treatment-intensity classification were based on the 2025 Global Initiative for Asthma (GINA) recommendations [32]. Asthma treatment intensity was categorized according to GINA treatment Steps 1–4 and analyzed as an ordinal variable. These GINA categories were used as an available treatment-intensity classification from the archived records and were not interpreted as an independent biomarker-based severity endotype. For allergic rhinitis, diagnosis and severity classification followed the Allergic Rhinitis and its Impact on Asthma (ARIA) framework [33], using the established intermittent/persistent and mild/moderate-to-severe classification. Specifically, category 1 represented mild intermittent; category 2, moderate-to-severe intermittent; category 3, mild persistent; and category 4, moderate-to-severe persistent allergic rhinitis. In patients with concomitant allergic asthma and rhinitis, the ordinal asthma component was represented by GINA treatment Steps 2–4. Recorded variables included sex, body-mass category, smoking status, family history of allergic disease, disease duration, disease-specific severity/treatment-intensity category, sensitization profile, associated allergic conjunctivitis, and residential environment.

Available treatment variables included inhaled corticosteroid/LABA therapy, intranasal corticosteroids, antihistamines, leukotriene receptor antagonists, methylxanthines, and recorded corticosteroid use, depending on phenotype. No participant was receiving biologic therapy. Detailed standardized information on recent exacerbations, acute infections, or recent medication changes immediately before blood sampling was not available in the archived records.

### 2.3. Serum Interleukin-11 Measurement

Serum IL-11 concentrations were measured using the Quantikine Human IL-11 ELISA Kit (R&D Systems, Minneapolis, MN, USA; catalog no. D1100), based on the quantitative sandwich ELISA principle and performed according to the manufacturer’s instructions. Previously collected serum samples were stored at −80 °C until IL-11 measurement. According to the manufacturer, the minimum detectable dose (MDD) of IL-11 is typically <8.0 pg/mL, and the standard curve range is 31.2–2000 pg/mL. Manufacturer-reported intra-assay coefficients of variation for serum/plasma samples range from 4.4% to 4.8%, and inter-assay coefficients of variation range from 3.7% to 8.0%. Samples in the present study were analyzed as single determinations; therefore, study-specific replicate-based intra-assay CVs were not available. Values below the assay detection limit were recorded in the archived dataset as 0.00 pg/mL and represent below-detection-limit observations rather than true zero concentrations. A separate validated lower limit of quantification (LOQ) was not specified in the available manufacturer documentation and was not retrospectively inferred. Concentrations were expressed in pg/mL. Detailed archived records regarding time-to-centrifugation, the exact number of freeze–thaw cycles, formal hemolysis/lipemia assessment, and ELISA plate/batch allocation were not available for retrospective analysis. Therefore, potential plate- or batch-related effects could not be formally assessed or adjusted for.

### 2.4. Statistical Analysis

The archived individual-level dataset was reanalyzed using Python version 3.13.5 (Python Software Foundation, Wilmington, DE, USA), pandas version 2.2.3, SciPy version 1.17.0, and statsmodels version 0.14.6. Serum IL-11 distributions were summarized using mean, standard deviation (SD), median, interquartile range (IQR), and observed minimum–maximum range. Because the IL-11 distributions were broad and included observations recorded as 0.00 pg/mL for concentrations below the assay detection limit, the primary global comparison used the Kruskal–Wallis test. Because values recorded as 0.00 pg/mL represented concentrations below the assay detection limit, a sensitivity analysis was additionally performed in which these observations were replaced by 4.0 pg/mL, corresponding to one-half of the manufacturer-reported upper bound of the typical MDD (<8.0 pg/mL). Pairwise comparisons were conducted using two-sided Mann–Whitney U tests with Holm adjustment for multiple testing. Effect sizes for pairwise non-parametric comparisons were quantified using Cliff’s delta, with 95% confidence intervals estimated using 10,000 bootstrap resamples with a fixed random seed of 42. The magnitude of the global Kruskal–Wallis effect was additionally summarized using epsilon-squared. Confidence intervals for effect sizes were exploratory and were not multiplicity-adjusted. One-way analysis of variance was used as a sensitivity analysis.

Associations between serum IL-11 and disease-specific ordinal categories were evaluated separately within each clinical phenotype using Spearman rank correlation. Numerical IL-11 distributions were also summarized for each available disease-specific category. For asthma phenotypes, the ordinal variable corresponded to GINA treatment Steps, whereas for allergic rhinitis it corresponded to ARIA severity categories. Because the study groups differed with respect to age, sex, body-mass category, and smoking status, an exploratory covariate-adjusted sensitivity analysis was performed. Serum IL-11 concentration was modeled using ordinary least-squares linear regression according to clinical phenotype, with healthy controls as the reference group, while simultaneously adjusting for age, sex, body-mass category, and current smoking status. Robust standard errors were used because of the modest sample size and potential heteroscedasticity. Given the relatively small and uneven phenotype-specific groups, this model was considered exploratory and was not used to replace the prespecified non-parametric primary analysis. All tests were two-sided, and *p* < 0.05 was considered statistically significant. The analyses were exploratory, and no a priori clinically meaningful difference for serum IL-11 had been established. To characterize the sensitivity of the available sample, a minimum detectable effect-size calculation was performed for the four-group comparison. With *n* = 88, four groups, α = 0.05, and 80% power, the corresponding detectable standardized effect was approximately Cohen’s f = 0.36. Thus, the study was capable of detecting moderate-to-large overall between-group effects, whereas smaller effects may have remained undetected.

### 2.5. Ethics Approval and Informed Consent

Given the retrospective design and the use of fully anonymized archived clinical data and previously collected serum samples, formal ethics committee approval was not required according to the applicable institutional and national regulations. The study was conducted in accordance with the Declaration of Helsinki. Written informed consent for participation was obtained from all participants.

## 3. Results

### 3.1. Study Population

The study included 88 adults: 31 with allergic rhinitis, 15 with non-allergic asthma, 22 with allergic asthma associated with allergic rhinitis, and 20 healthy controls. Baseline demographic and clinical characteristics are summarized in Table 1. The groups were not fully comparable with respect to baseline demographic characteristics. Participants with non-allergic asthma were older than those in the other groups, while healthy controls showed a marked female predominance. Body-mass category distributions also differed across phenotypes, with obesity being more frequent in the non-allergic asthma group and most healthy controls being of normal weight. Current smoking was present in 1/15 (6.7%) participants with non-allergic asthma, 4/31 (12.9%) with allergic rhinitis, none of the 22 participants with allergic asthma and rhinitis, and 5/20 (25.0%) healthy controls.

Among the 53 participants with allergic disease, 14 (26.4%) had seasonal-only sensitization, 20 (37.7%) had perennial-only sensitization, and 19 (35.8%) had mixed seasonal and perennial sensitization. In the allergic rhinitis group, 14/31 (45.2%) had seasonal-only sensitization, 7/31 (22.6%) had perennial-only sensitization, and 10/31 (32.3%) had mixed sensitization. In the allergic asthma associated with allergic rhinitis group, 13/22 (59.1%) had perennial-only sensitization and 9/22 (40.9%) had mixed sensitization; no participant in this group had seasonal-only sensitization.

Regarding specific aeroallergen categories, pollen sensitization was present in 24/31 (77.4%) participants with allergic rhinitis and 9/22 (40.9%) with allergic asthma associated with rhinitis, whereas sensitization to house-dust-related allergens was present in 15/31 (48.4%) and 21/22 (95.5%), respectively. Mold sensitization was recorded in 6/22 (27.3%) participants with allergic asthma associated with rhinitis and in none of those with allergic rhinitis. Sensitization to cat or dog allergens was uncommon in both groups.

In the non-allergic asthma group, the numbers of participants in GINA Steps 1, 2, 3, and 4 were 4, 5, 3, and 3, respectively. In the allergic rhinitis group, the numbers in ARIA categories 1–4 were 2, 2, 22, and 5. In the allergic asthma with rhinitis group, GINA Steps 2, 3, and 4 included 9, 8, and 5 participants, respectively.

### 3.2. Serum IL-11 Distribution Across Study Groups

**Figure 1:**
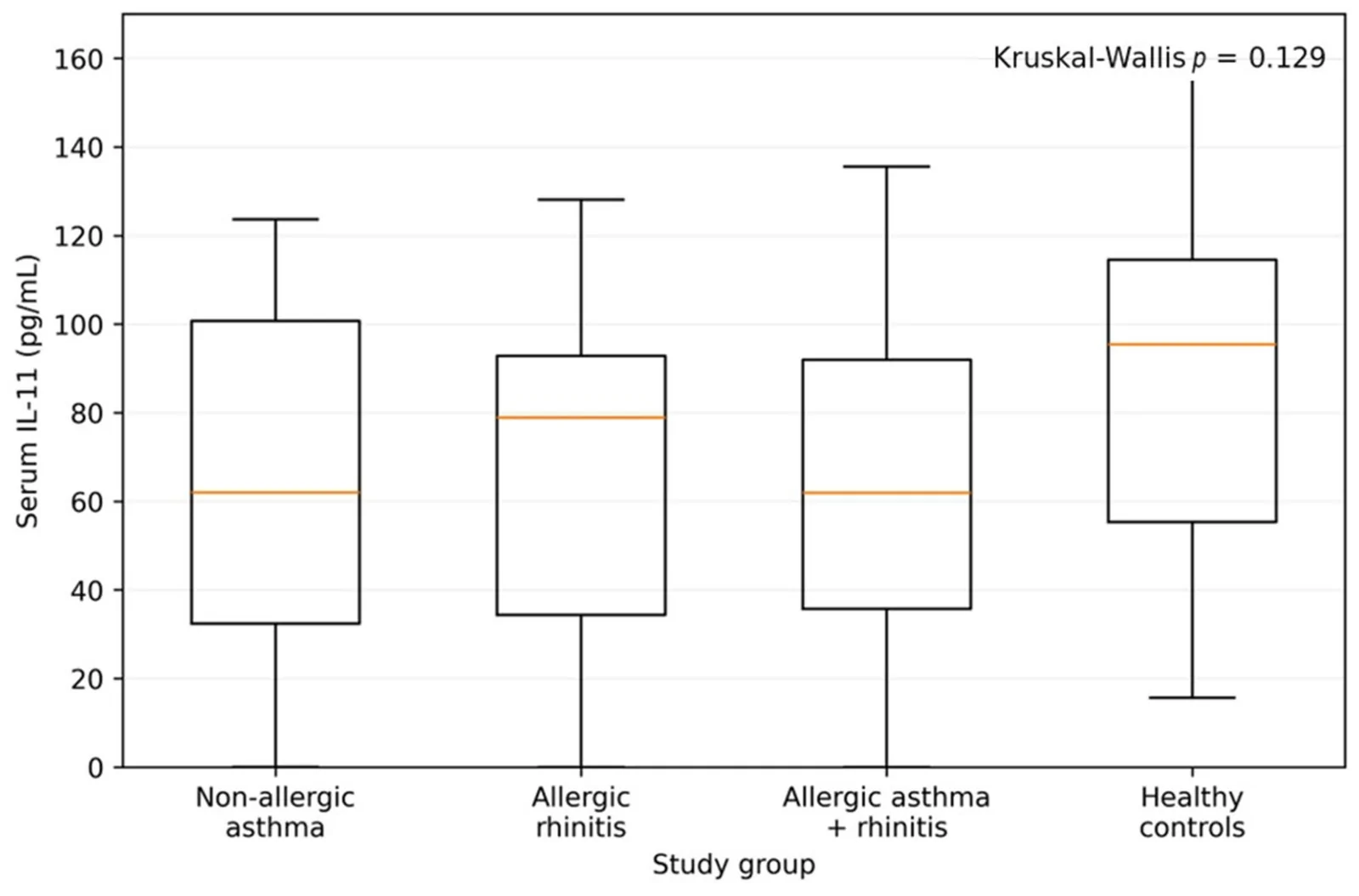
Distribution of serum IL-11 concentrations across the four study groups. Boxes represent the interquartile range, center lines indicate medians, and whiskers indicate the observed minimum–maximum range. The global Kruskal-Wallis test was not significant (*p* = 0.129).

Serum IL-11 concentrations showed broad overlap across the three airway disease phenotypes and healthy controls (Table 2 and Figure 1). Mean concentrations were 63.98 pg/mL in non-allergic asthma, 65.11 pg/mL in allergic rhinitis, 64.12 pg/mL in allergic asthma associated with rhinitis, and 89.01 pg/mL in healthy controls. Median concentrations were 62.01, 78.87, 61.92, and 95.46 pg/mL, respectively.

The global Kruskal-Wallis comparison was not statistically significant (H = 5.667, *p* = 0.129). The one-way ANOVA sensitivity analysis yielded a concordant result (F = 1.922, *p* = 0.132). No pairwise comparison remained statistically significant after Holm correction. The overall Kruskal–Wallis effect size was small (epsilon-squared = 0.032). Pairwise Cliff’s delta estimates were close to zero for comparisons among the three clinical phenotypes: non-allergic asthma versus allergic rhinitis, δ = 0.015 (95% CI −0.351 to 0.368); non-allergic asthma versus allergic asthma with rhinitis, δ = 0.006 (95% CI −0.376 to 0.388); and allergic rhinitis versus allergic asthma with rhinitis, δ = 0.019 (95% CI −0.302 to 0.346). Comparisons with healthy controls showed larger negative effect estimates: non-allergic asthma versus controls, δ = −0.380 (95% CI −0.720 to −0.007); allergic rhinitis versus controls, δ = −0.348 (95% CI −0.632 to −0.039); and allergic asthma with rhinitis versus controls, δ = −0.336 (95% CI −0.659 to 0.009). These confidence intervals were wide, and none of the corresponding pairwise comparisons remained statistically significant after Holm correction. Thus, circulating IL-11 did not differentiate the three airway disease phenotypes from healthy controls in this cohort.

Six of 88 IL-11 measurements (6.8%) were below the assay detection limit, including three in the allergic rhinitis group and three in the allergic asthma with rhinitis group. No below-detection-limit observations occurred in the non-allergic asthma or healthy control groups. In a sensitivity analysis replacing these observations with 4.0 pg/mL, corresponding to one-half of the manufacturer-reported upper bound of the typical MDD (<8.0 pg/mL), the overall result remained virtually unchanged (Kruskal–Wallis H = 5.671, *p* = 0.129). The corresponding one-way ANOVA also remained non-significant (F = 1.911, *p* = 0.134), indicating that the primary conclusion was robust to this alternative handling of below-detection-limit values.

An exploratory covariate-adjusted analysis addressing baseline demographic imbalance is reported in Section 3.4.

Values recorded as 0.00 pg/mL in the archived dataset represented concentrations below the assay detection limit and are reported as <LOD in the observed ranges.

### 3.3. Exploratory Association with Disease-Specific Clinical Categories

Serum IL-11 was not significantly associated with GINA treatment step in non-allergic asthma (Spearman rho = −0.170, *p* = 0.544), with ARIA severity category in allergic rhinitis (rho = 0.046, *p* = 0.805), or with GINA treatment step in allergic asthma associated with allergic rhinitis (rho = 0.052, *p* = 0.818). Numerical values by disease-specific ordinal category are shown in Table 3. No consistent monotonic trend across the corresponding disease-specific categories was observed within any of the three clinical phenotypes.

### 3.4. Exploratory Covariate-Adjusted Analysis

Because the study groups differed with respect to age, sex, body-mass category, and smoking status, an exploratory covariate-adjusted analysis was performed. Female sex was not independently associated with serum IL-11 concentration in the same adjusted model (β = −14.59 pg/mL, 95% CI −34.17 to 4.98, *p* = 0.144). In the overall cohort, median IL-11 concentrations were 72.64 pg/mL in women and 86.50 pg/mL in men (Mann–Whitney *p* = 0.736). In an ordinary least-squares linear regression model with robust standard errors, healthy controls were used as the reference group and age, sex, body-mass category, and current smoking status were included simultaneously as covariates. After adjustment, serum IL-11 concentrations were lower in the non-allergic asthma group (β = −41.99 pg/mL, *p* = 0.023), allergic rhinitis group (β = −32.37 pg/mL, *p* = 0.010), and allergic asthma with rhinitis group (β = −33.51 pg/mL, *p* = 0.022) compared with healthy controls. Because of the modest and uneven group sizes and baseline imbalance, these findings were considered exploratory and hypothesis-generating rather than confirmatory.

## 4. Discussion

The principal finding of this exploratory study was that circulating IL-11 concentrations did not differ significantly among adults with allergic rhinitis, non-allergic asthma, allergic asthma associated with allergic rhinitis, and healthy controls. In addition, serum IL-11 showed no significant monotonic association with the corresponding disease-specific ordinal clinical category within any of the three airway disease phenotypes. These findings do not challenge the established biological relevance of IL-11 in airway injury, inflammation, and remodeling; rather, they suggest that a single circulating measurement may not adequately reflect local airway IL-11 activity or provide sufficient discriminatory value as a stand-alone clinical biomarker. The close clinical and pathophysiological relationship between allergic rhinitis and asthma is well established, supporting their evaluation within the united-airways framework [34,35,36,37].

The negative circulating-biomarker finding is informative when considered alongside tissue-based and experimental evidence. Increased IL-11 expression has been demonstrated in bronchial tissue from patients with more severe asthma, while targeted IL-11 expression in experimental airway models has been associated with lymphocytic inflammation, subepithelial fibrosis, smooth-muscle alterations, extracellular-matrix deposition, and airway obstruction. Subsequent studies have further supported an important role for IL-11 in fibroblast activation, pulmonary fibrosis, epithelial dysfunction, and impaired tissue regeneration. However, local tissue expression and systemic circulating concentrations represent distinct biological compartments. Accordingly, the present findings should be interpreted as an assessment of circulating IL-11 biomarker performance and not as evidence against a biological role of IL-11 within airway tissue.

Several mechanisms may explain the absence of a detectable serum signal. IL-11 may act predominantly through local paracrine signaling, with limited release into the systemic circulation. Serum concentrations may additionally be influenced by clearance, binding proteins, intermittent production, treatment exposure, and non-airway sources. Moreover, circulating IL-11 may not adequately reflect structural airway remodeling, which develops over time and may be more closely related to tissue expression, pulmonary-function indices, extracellular-matrix biomarkers, or imaging-derived measures.

The numerically higher IL-11 concentrations observed among healthy controls were not statistically significant in the primary unadjusted global analysis or after multiplicity-adjusted pairwise comparisons. However, in an exploratory model adjusted for age, sex, body-mass category, and smoking status, IL-11 concentrations were significantly lower in each clinical phenotype relative to healthy controls. Given the modest group sizes, baseline imbalance, and inability to account for potentially relevant pre-analytical factors, this post hoc adjusted finding should be regarded as hypothesis-generating rather than evidence of a protective or inverse biological relationship. The broad overlap among groups and the absence of a consistent severity gradient indicate limited discriminatory performance in this dataset. These findings emphasize the importance of appropriate control groups, transparent reporting of negative biomarker results, and careful separation of mechanistic plausibility from clinical biomarker performance. More broadly, experience from other areas of clinical allergology also illustrates that individual diagnostic tests may not always reflect the underlying clinical phenotype, emphasizing the importance of interpreting isolated laboratory or diagnostic findings within the broader clinical context [38].

The inclusion of isolated allergic rhinitis is relevant within the united-airways framework. Although IL-11 has been extensively investigated in fibrosis and increasingly in lower-airway disease, evidence regarding its circulating concentrations in allergic rhinitis remains limited. Our findings suggest that serum IL-11 does not provide a simple systemic biomarker linking upper- and lower-airway disease phenotypes, at least when assessed at a single time point in a modest single-center cohort.

From a clinical perspective, serum IL-11 should not currently be considered an isolated marker of airway disease presence or severity. Future studies should prioritize paired systemic and local-airway measurements, longitudinal sampling, pulmonary-function parameters, disease-control scores, exacerbation history, treatment exposure, and markers of extracellular-matrix remodeling or epithelial injury. Multimarker strategies may ultimately prove more informative than assessment of IL-11 alone.

Recent deep-phenotyping frameworks in asthma further emphasize the integration of molecular, metabolic, and neuro-hormonal endotypes to improve precision assessment beyond isolated biomarkers [39].

The study evaluated three clinically distinct airway disease phenotypes and healthy controls within the same center and used the same serum assay for all participants. Reanalysis of the individual-level dataset enabled transparent reporting of distributions, ranges, multiplicity-adjusted comparisons, and phenotype-specific severity analyses.

Several limitations should be acknowledged. The retrospective single-center design and modest phenotype-specific sample sizes limit the generalizability of the findings. Some severity strata included only a small number of participants, and serum IL-11 was measured at a single time point. A single cytokine measurement cannot capture short-term biological variability or establish temporal stability. Because the archived records did not systematically document whether sampling occurred during a clinically stable period, transient effects related to exacerbation, infection, recent allergen exposure, or treatment changes may have contributed to the observed within-group variability. In addition, the study groups were not fully matched with respect to age, sex, body-mass category, and smoking status. Although the available baseline variables were incorporated into an exploratory adjusted model, the retrospective dataset did not permit comprehensive standardized characterization of all potentially relevant comorbidities, and the modest sample size limited the stability and completeness of covariate adjustment. Therefore, residual confounding by unmeasured clinical factors cannot be excluded. The absence of complete eosinophil, total IgE, FeNO, and airway inflammatory-cell data also prevented more detailed inflammatory endotyping within the asthma phenotypes. Accordingly, the present study cannot assess whether circulating IL-11 is related to classical type 2 inflammatory markers or to specific inflammatory endotypes. The absence of systematically available spirometric parameters and standardized asthma-control scores also prevented assessment of the relationship between circulating IL-11 and objective airflow limitation or current disease control. The marked female predominance in the healthy control group is particularly relevant because this group also showed the highest unadjusted IL-11 concentrations. Although sex was not independently associated with IL-11 in the exploratory adjusted model, residual confounding related to the unequal sex distribution cannot be excluded, and phenotype-specific sex-stratified analyses were limited by the small numbers within several strata. Because healthy controls were not characterized using a uniform prospective respiratory and allergy screening protocol, subclinical or undocumented airway disease or atopy cannot be completely excluded. This introduces a potential risk of control-group misclassification and may have contributed to the observed variability in circulating IL-11 concentrations.

Detailed pre-analytical metadata, including time-to-centrifugation, freeze–thaw history, formal assessment of hemolysis/lipemia, and ELISA plate/batch allocation, were not available in the archived dataset. Accordingly, residual pre-analytical or batch-related variability cannot be excluded.

The study was exploratory and was not prospectively powered using an established clinically meaningful serum IL-11 difference. Given the available sample size, the study was capable of detecting approximately moderate-to-large overall group effects (Cohen’s f ≈ 0.36 at 80% power), whereas smaller effects may have remained undetected. Because no validated clinically meaningful or equivalence margin for circulating IL-11 was available, the absence of statistical significance in the primary comparison should not be interpreted as evidence of equivalence between groups.

The archived records did not permit standardized assessment of recent exacerbations, intercurrent infections, or recent medication changes before blood sampling. These factors, as well as differences in conventional treatment exposure, may have influenced circulating IL-11 concentrations and represent potential residual confounders. Because treatment dose, duration, adherence, and the timing of systemic corticosteroid exposure relative to blood sampling were not available in a standardized form, treatment-related effects on circulating IL-11 could not be fully assessed.

Pulmonary-function indices, disease-control scores, standardized longitudinal exacerbation data, systematically available inflammatory biomarkers such as CRP, IL-6, peripheral blood eosinophil counts, FeNO, and periostin, local airway measurements, and structural remodeling markers were not available. Therefore, the present study cannot exclude a local, treatment-related, or longitudinal relationship between IL-11 and airway pathology. The findings should be interpreted as an exploratory assessment of circulating IL-11 rather than as a definitive evaluation of IL-11 biology in airway disease.

## 5. Conclusions

In the primary unadjusted analysis, circulating IL-11 concentrations did not differ significantly among adults with allergic rhinitis, non-allergic asthma, allergic asthma associated with allergic rhinitis, and healthy controls. Serum IL-11 was also not significantly associated with GINA treatment step in the asthma phenotypes or ARIA severity category in allergic rhinitis. An exploratory covariate-adjusted analysis suggested lower circulating IL-11 concentrations in each clinical phenotype relative to healthy controls; however, given the modest group sizes and baseline imbalance, this finding should be regarded as hypothesis-generating. In this exploratory single-center cohort, a single serum IL-11 measurement did not demonstrate sufficient discriminatory ability to distinguish the studied airway disease phenotypes or their disease-specific clinical categories. These findings should not be interpreted as definitively excluding a potential biomarker role for circulating IL-11. Prospective studies combining local-airway and systemic measurements in larger, well-balanced cohorts are required to clarify its potential clinical relevance.

## Figures and Tables

**Table 1:** Baseline demographic and clinical characteristics of the study population.

Characteristic	Non-Allergic Asthma (*n* = 15)	Allergic Rhinitis (*n* = 31)	Allergic Asthma + Rhinitis (*n* = 22)	Healthy Controls (*n* = 20)
Age, years, mean ± SD	46.2 ± 16.6	28.5 ± 8.2	29.8 ± 12.1	25.1 ± 4.9
Age, median [IQR]	52.0 [33.5–60.0]	27.0 [20.0–33.5]	25.5 [20.0–32.5]	23.0 [21.0–29.3]
Female sex, *n* (%)	7 (46.7%)	14 (45.2%)	12 (54.5%)	17 (85.0%)
Current smokers, *n* (%)	1 (6.7%)	4 (12.9%)	0 (0.0%)	5 (25.0%)
Normal weight, *n* (%)	3 (20.0%)	20 (64.5%)	13 (59.1%)	19 (95.0%)
Overweight, *n* (%)	4 (26.7%)	10 (32.3%)	5 (22.7%)	0 (0.0%)
Obesity grade 1, *n* (%)	6 (40.0%)	0 (0.0%)	3 (13.6%)	1 (5.0%)
Obesity grade 2, *n* (%)	1 (6.7%)	1 (3.2%)	0 (0.0%)	0 (0.0%)
Obesity grade 3, *n* (%)	1 (6.7%)	0 (0.0%)	1 (4.5%)	0 (0.0%)
ICS/LABA therapy, *n* (%)	10 (66.7%)	0 (0.0%)	10 (45.5%)	0 (0.0%)
Inhaled corticosteroid therapy, *n* (%)	6 (40.0%)	0 (0.0%)	0 (0.0%)	0 (0.0%)
Antihistamines, *n* (%)	2 (13.3%)	31 (100%)	15 (68.2%)	0 (0.0%)
Intranasal corticosteroids, *n* (%)	3 (20.0%)	15 (48.4%)	1 (4.5%)	0 (0.0%)
Leukotriene receptor antagonists, *n* (%)	1 (6.7%)	3 (9.7%)	11 (50.0%)	0 (0.0%)
Methylxanthines, *n* (%)	3 (20.0%)	0 (0.0%)	1 (4.5%)	0 (0.0%)
Biologic therapy, *n* (%)	0 (0.0%)	0 (0.0%)	0 (0.0%)	0 (0.0%)

Data are presented as mean ± SD, median [IQR], or number (%), as appropriate. Note: Treatment categories were not mutually exclusive; individual participants could receive more than one medication class. Treatment information reflects medication use recorded in the archived dataset. Detailed standardized information regarding dose, duration, adherence, and recent treatment changes was not consistently available.

**Table 2:** Distribution of serum IL-11 concentrations across study groups.

Group	*n*	Mean ± SD (pg/mL)	Median (pg/mL)	IQR (pg/mL)	Range (pg/mL)
Non-allergic asthma	15	63.98 ± 39.20	62.01	32.37–100.72	0.04–123.70
Allergic rhinitis	31	65.11 ± 40.46	78.87	34.38–92.86	<LOD–128.08
Allergic asthma + allergic rhinitis	22	64.12 ± 42.55	61.92	35.74–91.97	<LOD–135.54
Healthy controls	20	89.01 ± 37.31	95.46	55.32–114.53	15.66–155.97

**Table 3:** Serum IL-11 concentrations by phenotype and disease-specific ordinal category.

Phenotype	Disease-Specific Category	*n*	Mean (pg/mL)	SD (pg/mL)	Range (pg/mL)
Non-allergic asthma	GINA Step 1	4	67.10	33.73	25.70–107.57
Non-allergic asthma	GINA Step 2	5	69.85	36.63	28.25–113.33
Non-allergic asthma	GINA Step 3	3	84.17	56.70	19.21–123.70
Non-allergic asthma	GINA Step 4	3	29.82	26.11	0.04–48.79
Allergic rhinitis	ARIA category 1 (mild intermittent)	2	79.85	65.80	33.33–126.38
Allergic rhinitis	ARIA category 2 (moderate-to-severe intermittent)	2	38.19	0.66	37.72–38.66
Allergic rhinitis	ARIA category 3 (mild persistent)	22	65.02	41.20	<LOD–128.08
Allergic rhinitis	ARIA category 4 (moderate-to-severe persistent)	5	70.38	42.18	<LOD–107.33
Allergic asthma + allergic rhinitis	GINA Step 2	9	57.67	40.24	<LOD–132.30
Allergic asthma + allergic rhinitis	GINA Step 3	8	73.31	36.48	<LOD–128.16
Allergic asthma + allergic rhinitis	GINA Step 4	5	61.02	60.50	<LOD–135.54

Note: For allergic rhinitis, ordinal categories were defined according to the ARIA framework as follows: category 1, mild intermittent; category 2, moderate-to-severe intermittent; category 3, mild persistent; and category 4, moderate-to-severe persistent disease. For asthma phenotypes, the ordinal categories corresponded to GINA treatment Steps. Values recorded as 0.00 pg/mL in the archived dataset represented concentrations below the assay detection limit and are therefore shown as <LOD in the observed ranges. Because several strata contained small numbers of participants, category-specific values should be interpreted descriptively.

## Data Availability

The anonymized data supporting the findings of this study are available from the corresponding authors upon reasonable request, subject to institutional and ethical restrictions.
